# Development of a Quantitative PCR Assay for Four Salmon Species Inhabiting the Yangyangnamdae River Using Environmental DNA

**DOI:** 10.3390/biology10090899

**Published:** 2021-09-11

**Authors:** Muhammad Hilman Fu’adil Amin, Ji-Hyun Lee, Ah Ran Kim, Ju-Kyoung Kim, Chung-Il Lee, Hyun-Woo Kim

**Affiliations:** 1 Industry 4.0 Convergence Bionics Engineering, Pukyong National University, Busan 48513, Korea; m-hilman-f-a@fst.unair.ac.id; 2 Advance Tropical Biodiversity, Genomics, and Conservation Research Group, Department of Biology, Faculty of Science and Technology, Universitas Airlangga, Surabaya 60115, Indonesia; 3 Department of Marine Biology, Pukyong National University, Busan 48513, Korea; jhlee208@pukyong.ac.kr; 4 Marine Integrated Biomedical Technology Center, The National Key Research Institutes in Universities, Pukyong National University, Busan 48513, Korea; ahrankim@pukyong.ac.kr; 5 Aquatic Living Resources Center of East Sea, Korea Fisheries Resources Agency (FIRA), Busan 46041, Korea; loginkjk@fira.or.kr or; 6 Department of Marine Bioscience, Gangneung-Wonju National University, Gangneung 25457, Korea; leeci@gwnu.ac.kr

**Keywords:** environmental DNA, biomonitoring, exotic species, native species, *Oncorhynchus*, qPCR

## Abstract

**Simple Summary:**

Regular surveys provide essential information to establish strategies for the effective conservation of salmon resources. As an alternative to conventional fish surveys, which are costly and laborious, quantitative PCR (qPCR) assays were established for the analysis of four salmon species inhabiting the Korean Peninsula. We designed four species-specific primer sets that showed high specificity and sensitivity in both tissue and environmental DNA (eDNA) samples collected from the Yangyangnamdae River. After normalization for PCR inhibition in each sample, the established qPCR assays produced standardized and realistic eDNA profiles for the four salmon species, suggesting that the newly developed qPCR assays are a useful tool for the management of *Oncorhynchus* resources in Korean waters.

**Abstract:**

A species-specific quantitative PCR (qPCR) assay using environmental DNA (eDNA) is a promising tool for both qualitative and quantitative analyses of target species directly from water samples. Despite its reliability, an eDNA-based qPCR assay pipeline has not yet developed to monitor salmon species inhabiting Korean waters, which have been rapidly decreasing. We designed species-specific primers for four *Oncorhynchus* species inhabiting the eastern coastal waters along the Korean Peninsula. These include primers for two native species (*Oncorhynchus keta* and *O. masou*) and two that were introduced (*O. mykiss* and *O. kisutch*). The limit of detection and limit of quantification for the four qPCR assays ranged from 4.11 to 10.38 copies and from 30 to 81 copies, respectively, indicating a high sensitivity and specificity across all four species. Following optimization, the qPCR assays were used for the quantitative analyses of the four *Oncorhynchus* species in the Yangyangnamdae River during the spawning and non-spawning seasons in the year 2019–2020, one of the main rivers where salmon migrate during the spawning season in Korea. The raw copy numbers in all of the examined samples were normalized by PCR inhibition rates to standardize and compare with other studies. Among the four *Oncorhynchus* species examined, the eDNA concentration of *O. keta* increased significantly (63.60-fold, *p* < 0.0001) during the spawning season (November) compared with that in the non-spawning season (March), suggesting that *O. keta* is the main salmon species migrating through the Yangyangnamdae River. In contrast, we did not detect any differences in eDNA concentration for the other three *Oncorhynchus* species between the spawning and non-spawning seasons, indicating that their presence does not alter during the year. Their eDNA concentration is also relatively low compared to *O. keta*, which suggests that small numbers of these three species are present in the river. Overall, these newly developed qPCR assays represent useful monitoring tools for the management of four salmon species in Korean waters.

## 1. Introduction

*Oncorhynchus* species are semelparous and diadromous fish, which are among the most important species in the North Pacific region in an ecological, economical, and societal context [1,2]. Therefore, most North Pacific countries have implemented stock enhancement programs to maintain the populations of salmon species. The Republic of Korea is also among these countries and has conducted an artificial propagation program for the chum salmon, *Oncorhynchus keta*, since the 1980s [3]. However, salmon populations in the wild keep decreasing due to the loss and fragmentation of habitat [4,5], pollution [6], overfishing [7], and climate change [8,9,10]. After more than 500 metric tons of salmon were caught in the mid to late 1990s in Korea, the catch decreased to an average of 200 metric tons from 1997, in spite of an increased annual release of the fry [11,12]. In 2019, the chum salmon catch has declined considerably to 129.72 metric tons, threatening its sustainability in Korean waters [13]. However, the main cause of their rapid decrease is still not clearly understood in Korea. Although it is generally known that the environmental conditions in which early life history stages are exposed to impact the survival and population of salmon species [14,15], to our knowledge no survey has been conducted in the Korean rivers to measure their populations during both juvenile and spawning periods. Besides *O. keta*, several other *Oncorhynchus* species, including Masu salmon (*O. masou*), the other native species in Korea, has a population which has also rapidly decreased [16]. Two other introduced species (*O. kisutch* and *O. mykiss*) have also been reported in Korean waters [17]. Those species may have a potential negative impact on the native fish populations and the ecosystem of Korean rivers [18,19]. Therefore, regular monitoring for the *Oncorhynchus* species should be conducted for their scientific management and conservation in Korean waters.

Periodic fish surveys by traditional monitoring programs have required intensive skilled labor, long observation times, and a large budget to obtain reliable data [20,21]. A molecular approach using environmental DNA (eDNA) has been introduced as a promising alternative to overcome the challenges of traditional surveys [22]. eDNA represents the trace genetic material shed by all organisms in their habitat, which can be retrieved directly from environmental samples, including soil, feces, and water. eDNA drifting in the water column can be collected, isolated, amplified, and sequenced to reveal a genetic footprint of targeted aquatic species, which can then be used to establish their spatiotemporal distribution [23,24,25,26]. eDNA has been used to investigate fish species, including *Misgurnus fossilis* [27], *Macquaria australasica* [28], *Pseudorasbora parva* [29], *Clupea harengus*, *Gadus morhua*, *Platichthys flesus*, *Pleuronectes platessa*, and *Scomber scombrus* [30]. Given its versatility, tracking eDNA provides information on the route of migratory fishes [31,32], habitat connectivity and occupancy [33,34], spawning events [35,36,37], and quantitative patterns [38]. This approach may also be used to estimate the abundance and biomass of fish species, which is represented by the eDNA concentration of each target species [39,40,41]. In fact, the abundance of eDNA exhibits a strong relationship to fish abundance recorded by traditional estimation surveys, such as mark–recapture sampling [42], electrofishing [43,44], and catch per unit effort using gillnet [45] or fyke nets [46]. In addition to its sensitivity and reliability, eDNA analysis has also been shown to be more energy and cost efficient than other methods used currently for salmon species [44].

There have been several previous studies with respect to PCR analysis using eDNA to detect *Oncorhynchus* species using various molecular markers. For example, a study by Tillotson et al. [25] established a PCR assay for *O. nerka* using the cytochrome c oxidase subunit III gene. Laramie et al. [47] used the cytochrome c oxidase subunit I (COI) gene to detect *O. tshawytscha* in the upper Columbia River. Alternatively, Shelton et al. [48] used the cytochrome oxidase III/NADH dehydrogenase 3 (COIII/ND3) gene to estimate the population of *O. tshawytscha* in Skagit Bay, WA, USA, whereas Minegishi et al. [49] utilized a mitochondrial control region to measure *O. keta* in Otsuchi Bay, Japan. For multiple-species analysis, Levi et al. [20] assessed the number of *O. nerka* and *O. kisutch* in Auke Creek (Alaska) using a COI region, which was initially developed by Rasmussen et al. [50] to detect both species in food samples. These studies have been conducted on species inhabiting North America, and little information exists regarding the salmonid species in Korean rivers. Since extremely low degrees of genetic variation are often identified among some regional salmon species, establishment of a pipeline to detect local/regional populations is essential.

Among the seven species belonging to the genus *Oncorhynchus* in the North Pacific, four are present in South Korean waters. Chum (*O. keta*) and masu salmon (*O. masou*) natively inhabit Korean waters [51], whereas coho salmon (*O. kisutch*) and steelhead trout (*O. mykiss*) are species that have been introduced [17]. The primary aim of this study was to establish a quantitative PCR (qPCR) assay method to estimate biomasses of the four salmonids using eDNA directly from water samples collected from Korean waters. First, species-specific primer sets were designed for each species that targeted the mitochondrial cytochrome b (cytb) gene, and the performance and reliability of the assays, including the limit of detection (LOD) and the limit of quantification (LOQ), were evaluated. We also introduced a standardized quantification method by normalizing the raw copy numbers obtained by qPCR to the degree of inhibition of the internal positive control (IPC) values. The copy numbers of each species were then measured from water samples collected at multiple sites along the Yangyangnamdae River during the 2019–2020 spawning and no-spawning seasons. This newly developed multispecies qPCR assay provides a valuable tool for the scientific management and conservation of Korean waters by detecting and estimating population of those salmon species from eDNA samples.

## 2. Materials and Methods

### 2.1. Target Species and Molecular Assay Development

The species-specific primers for the four *Oncorhynchus* species (*O. keta*, *O. masou*, *O. mykiss*, and *O. kisutch*) found in Korea were designed using a bioinformatic analysis (Table 1). The mitochondrial cytb gene from each species was selected for the design of primers and a probe because of its inter-species sequence variation and a large number of reference sequences in the database [52]. A total of 40, 8, 13, and 25 unique cytb haplotypes for *O. keta*, *O. kisutch*, *O. masou*, and *O. mykiss* were obtained, respectively, from the GenBank database (https://www.ncbi.nlm.nih.gov/genbank/ accessed on 21 February 2020) (Table A1 in Appendix A). These haplotypes were aligned using MAFFT software [53] and primers and a probe for each species were designed based on the Primer3 [54] program of Geneious Software V9.1.8 [55].

### 2.2. Water Sample Collection and Environmental DNA Extraction

**Figure 1:**
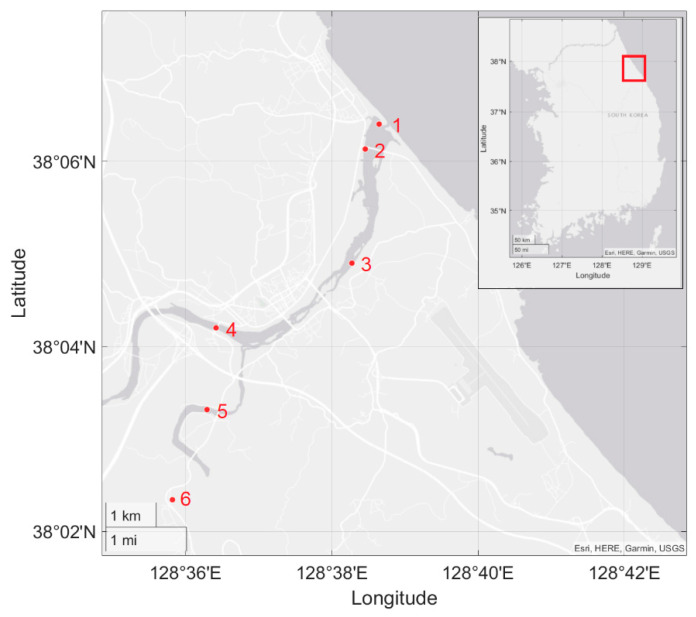
Six study sites in the Yangyangnamdae River, South Korea. Two tributaries, Hucheon and Namdae, were included.

Waters samples were collected from the surface and near the river center at six sites along the Yangyangnamdae River, Gangwon-do, Korea, where the Yangyang Inland Hatchery, the main salmon hatcheries in Korea, is located (Figure 1). This river is also the main river for returning salmon species to Korea [56]. Three sites (1, 2, and 3) were located along the main stream, while the remaining three sites (4, 5, and 6) were located along two tributaries, including Hucheon (site 4) and Namdae (site 5 and 6). Sample collections were conducted in November 2019 (spawning season) when the released salmon return to the river for spawning, and March 2020 (non-spawning season) [3]. Salinity was measured during water collection using a conductivity meter (CD-4307SD, Lutron Electronics, Coopersburg, PA, USA). Further, 1.5 L of water was collected from each site and immediately stored in ice until they were brought to the laboratory for filtration. Each 1.5 L water sample was split into three 500 mL sub-samples. Each sub-sample was subsequently shaken well before being filtered through a GN-6 Metricel membrane (PALL Life Sciences, Emiliano Zapata, Mexico). All of the glassware and filtration systems were treated with 10% commercial bleach containing 7.4% sodium hypochlorite before use. After filtration, the membranes were stored in 2.0 mL tubes in 630 µL of ATL buffer at −20 °C until DNA extraction. The filtered membrane containing ATL buffer was homogenized using a FastPrep-24™ Classic Instrument (MP Biomedicals, Irvine, CA, USA). The eDNA was extracted from homogenized membranes using DNeasy Blood and Tissue Kits (Qiagen GmBH, Hilden, Germany) according to the manufacturer’s instructions, and 50 µL of AE buffer was used for final elution. The extracted DNA was quantified with an ND-1000 NanoDrop (Thermo Scientific, Waltham, MA, USA), aliquoted, and stored at −20 °C until analysis.

### 2.3. Establishment of Quantitative PCR Assay

qPCR was conducted using all four species-specific primers together on the eDNA extracted from each sub-sample (three replicates per site). The predicted amplicon size for *O. keta,* *O. kisutch*, *O. masou*, and *O. mykiss* was 187, 138, 229, and 229 bp, respectively (Table 1). A 25 µL reaction mixture was prepared, and PCR conditions for each species are shown in Table A2. All of the PCRs were conducted using a Magnetic Induction Cycler system (Bio Molecular System, Upper Coomera, Australia). The amplified targets were cloned using the All in One^TM^ PCR Cloning Kit (BioFact, Daejeon, Korea), and the copies were quantified using a Quantus Fluorometer (Promega BioSystems, Sunnyvale, CA, USA). A standard curve was constructed using 10-fold serial dilutions of plasmids harboring each target sequence (from 1 to 10^−7^ ng). All of the PCRs were conducted with a negative control without template to monitor cross-contamination. Identical threshold levels of normalized fluorescence were applied in all assays to determine quantification cycle (Cq) values [57].

The specificity of the qPCR assays was evaluated by in silico analysis using GenBank Primer-BLAST [58] and validated by confirming the DNA sequence amplified by each species-specific primer set (Macrogen, Daejeon, Korea). To evaluate the sensitivity of our novel qPCR assays, the LOD and LOQ for each assay were calculated [59]. A 95% detection probability was designated for the LOD and a 35% coefficient of variation (C_V_) threshold for the LOQ [60,61]. C_V_s were fitted by best following models, exponential decay, linear, or polynomial models [62].

After normalization of the raw eDNA copy numbers using PCR inhibition rates determined by 2^ΔCq^ multiplication, the reactions were considered positive if the eDNA copy number was greater than that of the LOD [63], in at least one replicate per site [62]. Reactions were considered negative when the eDNA copy number was below the LOD value, whereas those with 0 < x < LOQ values were not used for further quantitative analysis. The eDNA copies per liter were calculated from copies per reaction (2 μL template volume) of the initial sample water (500 mL filtration volume and a 50 μL elution volume) using the equation established by Thomas et al. [64]. The log_10_(x + 1) transformation of eDNA copy number per liter was applied according to a previous eDNA study [65,66].

### 2.4. Inhibition Test

The inhibition of the PCRs by the eDNA samples was measured as described previously [67]. Primers targeting the ND2 gene of the exotic species, *Zenarchopterus dispar*, were used as an IPC (Table 1). The inhibition assay mixture (20 µL) consisted of 1× Luna^®^ Universal qPCR Master Mix (#M3003, New England Biolabs, Ipswich, MA, USA), 0.5 µM of each forward and reverse primer, 2 µL of IPC template, and 2 µL of extracted eDNA from each water sample. PCRs for the inhibition assay consisted of a 5 min initial denaturation at 95 °C, followed by 40 cycles of 95 °C for 20 s, annealing at 64 °C for 30 s, and extension at 72 °C for 20 s. Non-template assays were performed without IPC template. The PCR inhibition rate (ΔCq) was calculated by subtracting the Cq_sample_ from the Cq_positive control_. A shifted Cq value greater than three cycles of the IPC in the negative controls or non-amplification reactions was considered significant inhibition [68,69].

### 2.5. Data Analysis

A pairwise Wilcoxon rank-sum test was conducted to determine differences in eDNA concentration among salmon species, sampling events, and stream classification (mainstem and tributary). The statistical significance of ΔCq values between seasons was calculated using a *t*-test. The correlation between ΔCq and salinity was evaluated by Spearman’s rank correlation test. All statistical analyses were performed using the R statistical software V3.6.3 [70] and visualized using the ggplot2 package V3.3.5 [71].

## 3. Results

### 3.1. Profile and Performance of Primers and Probes

**Figure 2:**
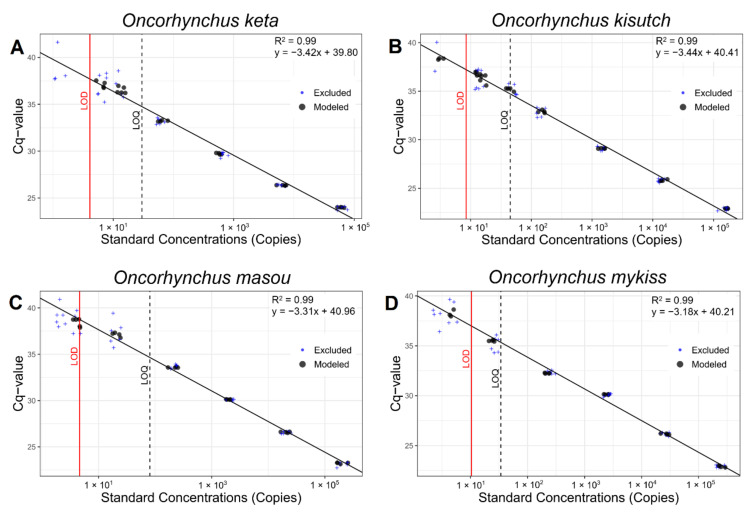
Calibration curves generated from serially diluted target samples using ten replications: (**A**) *O. keta*, (**B**) *O. kisutch*, (**C**) *O. masou*, and (**D**) *O. mykiss*. The vertical solid red and broken black line, respectively, represent the limit of detection (LOD) and the limit of quantification (LOQ).

**Figure A1:**
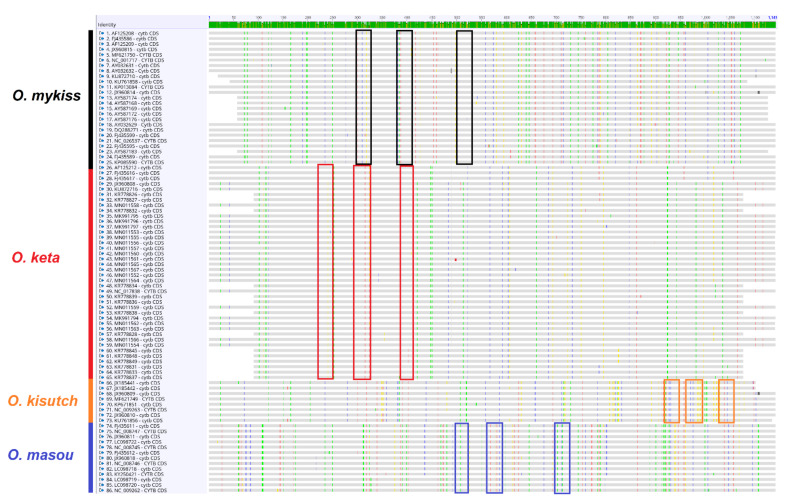
Multiple sequence alignment of primer regions from 86 haplotypes. Left, center, and right boxes indicate forward primer, probe, and reverse primer region, respectively. Each color represents the respective target salmon: *O. mykiss* (black), *O. keta* (red), *O. kisutch* (orange), and *O. masou* (blue).

**Figure A2:**
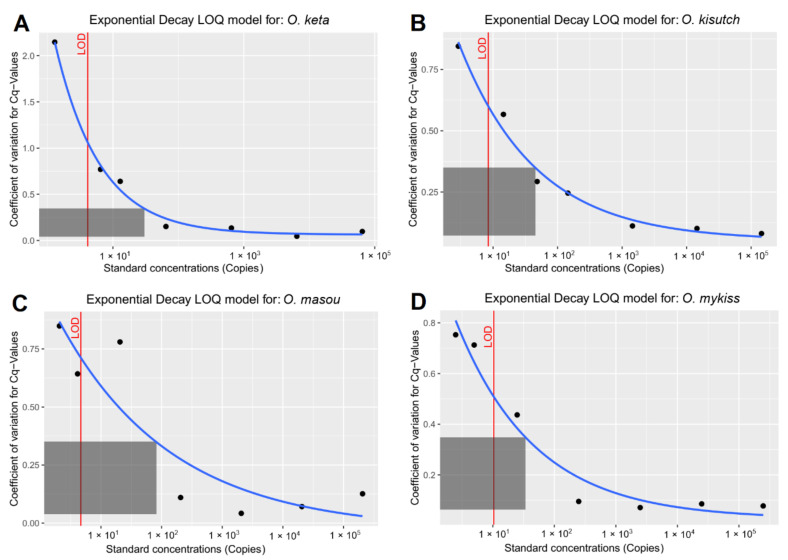
LOQ model for the respective salmon assays generated by plotting standard concentrations against the coefficient of variation (CV) of the Cq values. (**A**) *O. keta*, (**B**) *O. kisutch*, (**C**) *O. masou*, and (**D**) *O. mykiss* assay. All LOQs were determined by an exponential decay model. The LOD is presented as a vertical red line, the LOQ model is illustrated as a blue line, the gray area indicates 0.35 CV as a defined threshold of the LOQ, and the defined LOQ is determined by the conjoining of the LOQ model and the gray area of the CV.

The number of variable nucleotide sites ranged from four to nine in the primer region and five to seven in the probe, obtained by aligning 86 haplotypes of the four species (Figure A1). The DNA sequence of each amplicon also showed 100% identity to the reference sequence in the GenBank database. The LOD and LOQ were measured to assess the sensitivity of each assay (Figure 2, Table 2). The LOD values ranged from 4.11 copies (*O. keta*) to 10.38 copies (*O. mykiss*) with an average of 6.89 copies, which indicates a high degree of detection sensitivity in all four assays. LOQ values were obtained by an exponential decay model (Figure A2). The lowest and highest LOQ values were identified in *O. keta* (30 copies) and *O. masou* (81 copies), respectively. The PCR efficiency of all four assays was also high, and the values ranged from 95.15% in *O. kisutch* to 106.33% in *O. mykiss* with high r^2^ values (>0.99) (Table 2). Although the amplification rates were greater than 100% in the assays for *O. masou* (100.67%) and *O. mykiss* (106.33%), all of the values were within the accepted 10% variable range.

### 3.2. Inhibition in Environmental DNA Samples

**Figure 3:**
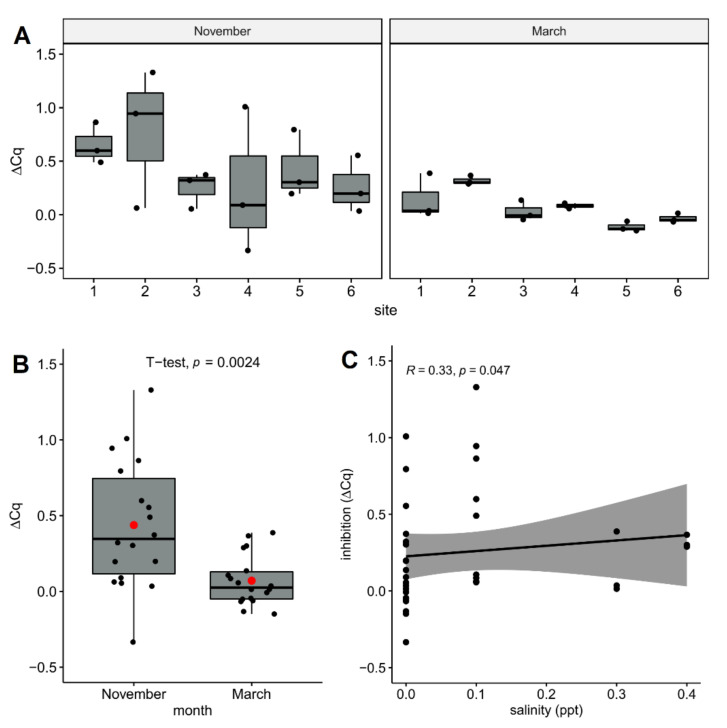
ΔCq value in the inhibition test of environmental DNA (eDNA) samples from the Yangyangnamdae River. (**A**) PCR inhibition across sampling sites and seasons; (**B**) mean and median values of delayed ΔCq value; and (**C**) Spearman correlation between ΔCq and salinity. Means and medians are presented as red circles and solid lines, respectively. Each data point is presented as a black dot.

All of the assays without the IPC templates exhibited negative amplification, demonstrating that IPC primers were not cross-reactive with eDNAs from the Yangyangnamdae River (data not shown). The average of ΔCq values (inhibition) was 0.25 and ranged from −0.33 at site four to 1.33 at site two in November. Compared with those in March (between −0.15 and 0.39), a much higher variation of inhibition was observed in November, in which values ranged from −0.33 to 1.33 (Figure 3A). The average inhibition in November (0.44 ± 0.42 cycles) were also higher (*p* < 0.005) than those in March (0.07 ± 0.16 cycles) (Figure 3B). Inhibition also exhibited a slight positive correlation (*p* = 0.047) with salinity (Figure 3C), suggesting that salinity is a factor that adversely affects eDNA amplification.

### 3.3. Environmental DNA Profiles in Field Testing

**Figure 4:**
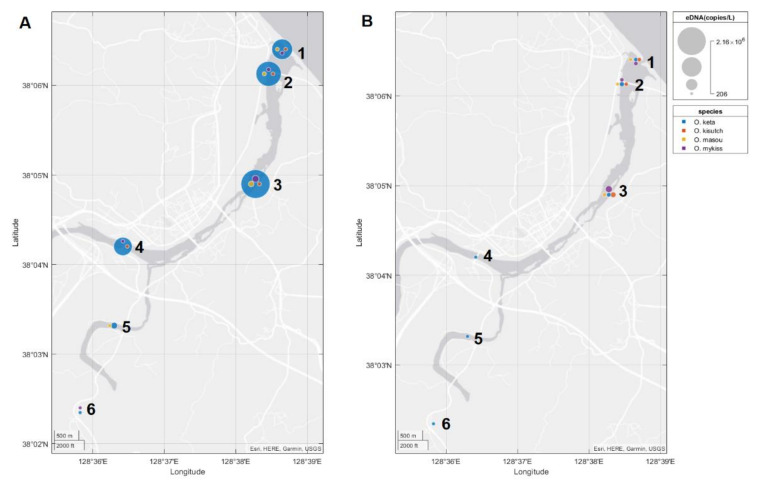
Mean eDNA concentration (copies/L) of four target salmon in the Yangyangnamdae River during the November (**A**) and March (**B**) sampling events. The sampling sites from the downstream are numbered.

*Oncorhynchus keta* DNA was detected across all six sites in both sampling events (Figure 4). The other three *Oncorhynchus* species were detected at both sampling events only in main stream. In tributaries, their detections were varied in November (Figure 4A), whereas no detection was observed for these species in March (Figure 4B). In November, *O. kisutch* was detected only in Hucheon tributary (site 4) and *O. masou* was detected in Namdae tributary (site five), while *O. mykiss* was detected in both tributaries (site four and six). A lack of amplification in all of the negative controls supported the accuracy of qPCR without any cross-contamination.

**Figure 5:**
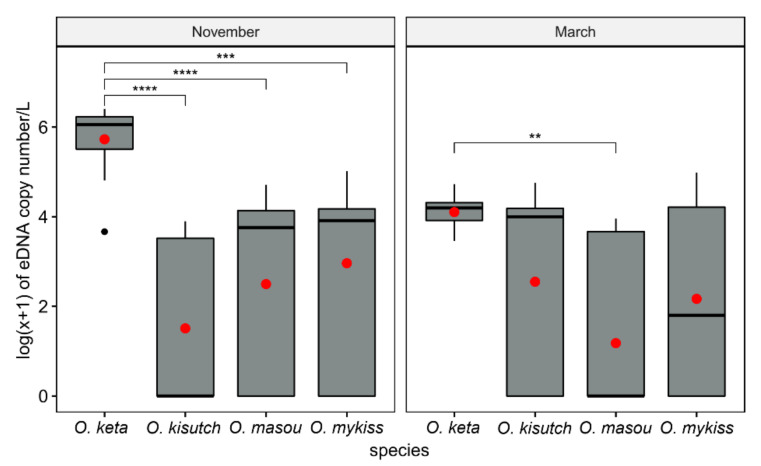
Log(x + 1) transformation of inhibition-normalized eDNA copy number per liter of four species of *Oncorhynchus* from the Yangyangnamdae River in November 2019 (spawning) and March 2020 (non-spawning) using whiskers plots. Means and medians are presented as red solid circles and horizontal solid lines within each box, respectively. Asterisks indicate the significance level (*p*) for each comparison by pairwise Wilcoxon rank-sum tests: ** *p* < 0.01; *** *p* < 0.001; **** *p* < 0.0001.

During the non-spawning season (March), the mean eDNA concentration of *O. keta* (1.71 × 10^4^ copies/L) was 8.70-fold higher (*p* < 0.01) than that of *O. masou* (1.96 × 10^3^ copies/L), whereas there was no statistical difference between the two other species, *O. kisutch* and *O. mykiss* (Figure 5). During the spawning season in November, the difference between *O. keta* and the other three species was significantly increased. The eDNA concentration values of *O. keta* were 626.10-, 77.11-, and 42.74-fold higher than those of *O. kisutch*, *O. masou*, and *O. mykiss*, respectively (Figure 5).

**Figure 6:**
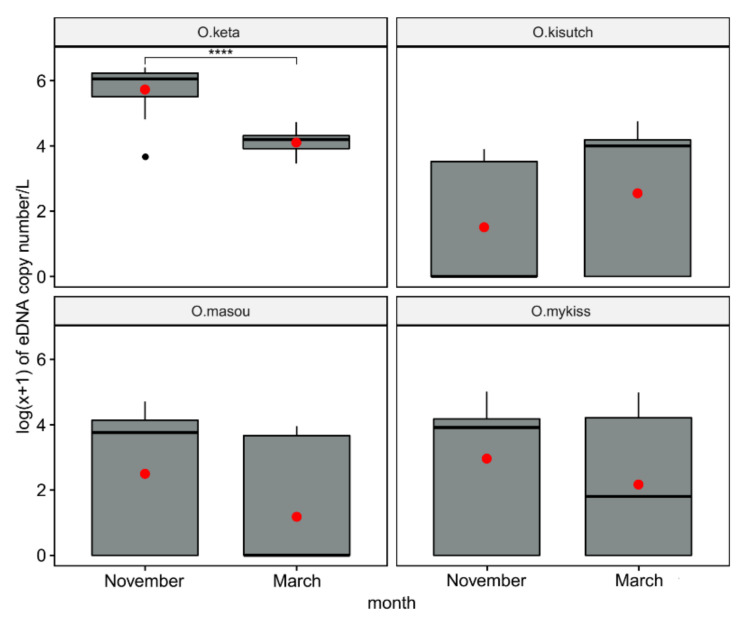
Log(x + 1) transformation of the inhibition-normalized eDNA copy number per liter of four species of *Oncorhynchus* in the Yangyangnamdae River compared by sampling event using whiskers plots. Means and medians are presented as red solid circles and horizontal solid lines within each box, respectively. Asterisks indicate the significance level (*p*) for each comparison by pairwise Wilcoxon rank-sum tests: **** *p* < 0.0001.

**Figure 7:**
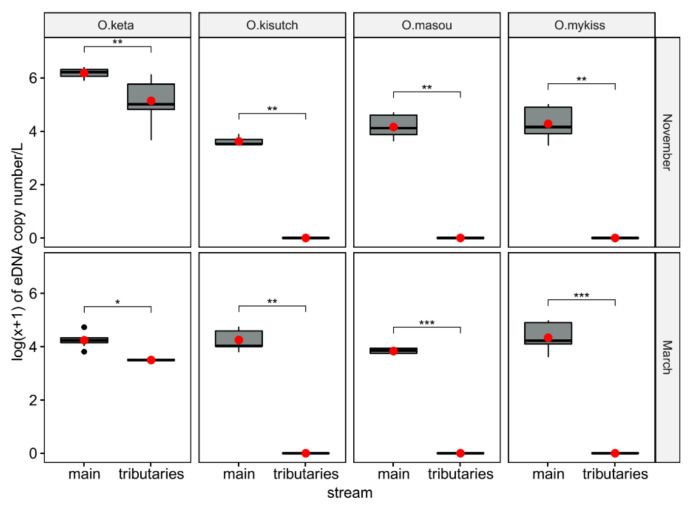
Log(x + 1) transformation of the inhibition-normalized eDNA copy number of the four species of *Oncorhynchus* in the Yangyangnamdae River compared between the main stream and tributaries using whiskers plots. Means and medians are presented as red circles and horizontal solid lines within each box, respectively. Asterisks indicate the significance level (*p*) for each comparison by pairwise Wilcoxon rank-sum tests: * *p* < 0.05; ** *p* < 0.01; *** *p* < 0.001.

The mean eDNA concentration of *O. keta* in November (1.09 × 10^6^ copies/L) was 63.60-fold higher (*p* < 0.0001) than that in March (1.71 × 10^4^ copies/L). Besides *O. keta*, there was no statistical difference in eDNA concentration of the other three *Oncorhynchus* species between March (non-spawning season) and November (spawning season) (Figure 6). Furthermore, eDNAs of all four *Oncorhynchus* species in the mainstream were significantly higher compared with those in tributaries during both sampling events, indicating a higher biomass of the salmon in the mainstream of the river (Figure 7). The ratio of *O. keta* between the mainstream and its tributaries decreased from 6.42 in March to 4.04 in November, reflecting a high number of catches at site three for artificial breeding (Figure 7).

## 4. Discussion

We successfully established qPCR assays for the four *Oncorhynchus* species that inhabit Korean rivers using the mitochondrial cytb gene. We then performed field studies to validate the application of these assays for determining eDNA quantities from environmental samples, indicating their reliability of use across various seasons and sampling sites. This will be a useful tool for the long-term monitoring of the two native salmon species in Korean rivers, providing an effective means of determining migration routes, seasonal changes in presence within the river system, as well quantifying the effectiveness of management programs or declines with future climate change. Additionally, it will also be useful in monitoring the two introduced salmon species in Korean waters, providing an opportunity to track future changes in their distribution, population size, and potential competition with native species.

The novel qPCR assays in our study exhibited high sensitivity, as evidenced by low mean LOD values (4.11 to 10.38 copies for each reaction). These values are close to the theoretically most sensitive LOD’s, three copies in each reaction, by assuming a 95% probability detection and a Poisson distribution [61]. Our study also revealed that triplicate detection at a single sampling site yielded an average copy number above the LOD, suggesting that the curve-fitting method defined by Merkes et al. [59] is reliable for determining and reporting the LOD and LOQ for eDNA-based surveillance. Non-modeled methods for determining the LOD and LOQ require a more refined range of standard concentrations, such as 1:2, to generate more accurate LOD and LOQ values, which requires additional time and cost [72]. Therefore, the effectiveness of eDNA surveys may be significantly reduced using non-modeled methods. Furthermore, the sensitivity test, including the LOD and LOQ determination, is required in the eDNA surveillance guidelines since many eDNA studies can detect low-abundance species within ecosystems. Previous species-specific eDNA studies in Pacific salmon do not contain adequate information regarding sensitivity tests, except that of Duda et al. [73], who studied five Pacific salmon in the Columbia River (USA). Xia et al. [74] reported that two of three eDNA studies consisted of newly developed markers, whereas only 88 of 165 studies determined the LOD of the marker. The evaluation of assay sensitivity helps in overcoming both overestimating and underestimating species-specific eDNA studies compared with traditional surveys. In the presence or absence of detection, normalized eDNA copy numbers below the LOD were excluded to avoid the risk of false-positive results. Although Klymus et al. [62] suggested that copy numbers below the LOD are acceptable as qualitative data in eDNA studies and are expected as very rare or low-abundance target species in the natural ecosystem, eDNA copies below the LOD result in inaccurate fish distribution and detection probabilities [75] because of the risk of a concentration plateau in PCR [76].

A high degree of variability in PCR inhibition were observed among the eDNA samples tested, with ΔCq values ranging from −0.33 to 1.33 cycles. This indicates that the copy numbers for each eDNA sample can be underestimated by up to 2.51-fold of the real values through PCR inhibition. Generally, more than three delayed cycles have been considered to be significant PCR inhibition, which is equivalent to a 10-fold underestimation in copy number [68]. A much higher degree of precision in the measurements is required for the quantitative analysis of fish eDNAs that exist in trace amounts in the water. Besides PCR inhibition, other factors during sample collection and DNA preparation may affect PCR results to cause inaccurate interpretations [77]. Our results indicate that the measurement of PCR inhibition should be considered for the more realistic quantification of salmon species regardless of sample sites or seasons. The normalization of raw copy numbers should be conducted to reduce at least one uncertainty, which would be helpful to obtain more accurate data in the quantitative analysis of eDNA. More importantly, the normalization of raw read numbers as “standardized values” is versatile and transformable to other studies across different research groups.

Interestingly, we identified a much higher degree of inhibition among the samples obtained in November compared with those in March. There are numerous potential PCR inhibitors in the environmental water samples, and it is not clear what was responsible for the PCR inhibition observed in November. One possible explanation would be that organic matter originating from leaves in the fall may be incorporated into the water stream. Organic acids from fallen leaves, such as humic, tannic, and phytic acids, are among well-known inhibitors of PCR [78,79]. We also identified higher PCR inhibition among sites in downstream areas compared with upstream regions and a positive correlation with salinity. Similarly, PCR inhibition has been detected previously in high-salinity environment, which inhibits the amplification of eDNA [80], making it imperative that salinity is recorded during all future sample collections.

Despite prospects of species-specific eDNA approach for aquatic species survey, several factors should be considered when adopting species-specific eDNA assays to other locations. For example, the assays should be re-validated when using different enzymes, master mixes, or qPCR instruments, including re-analyzing the LOD and LOQ values. Inhibition of eDNA amplification should also be tested at new sampling sites, even during different sampling events. In addition, eDNA behavior in the lotic habitat has an unexpected pattern of dispersion since several environmental characteristics affect transport, retention, and the dynamics of eDNA in the stream ecosystem, including water discharge [81,82], temperature [83], and substrate type [84,85]. Therefore, continued studies should be conducted to evaluate eDNA characteristics in streams and enhance the precision and interpretability of lotic eDNA results.

Among the two endemic species analyzed in this study, *O. keta* has been known to be the dominant species migrating through the Yangyangnamdae River [86,87,88]. According to the traditional net survey, the Yangyangnamdae River was the site containing more than 70% of *O. keta* returning to Korean waters beginning in late September, whereas most juveniles migrate to the East Sea before April [89,90]. These results are consistent with our current study in which the highest concentration of eDNA for *O. keta* was identified among the four examined species, especially at downstream areas in November. Since most of the returning individuals were caught at site three, where the hatchery is located, it is reasonable that the highest number of *O. keta* reside at sites downstream of the hatchery. However, *O. keta* eDNA was also detected upstream in November, indicating that some of the returning salmon could have escaped catch at site three and migrated upstream. Among the two tributaries, a higher *O. keta* eDNA concentration was identified at Hucheon, which is larger than the Namdae tributary. This result is inconsistent with a previous study in which *O. keta* were found in the Namdae tributary rather than in the Hucheon [89]. Many weirs and other artificial constructs have been built in the river, the impact of which have been well-studied in migratory fishes and found to be negative for anadromous species [91,92]. Further studies should be conducted to account for the differences in this study. The low degree of *O. keta* eDNAs throughout the sample sites in March may indicate that juveniles have already migrated to the East Sea, which would be earlier than that observed in previous studies [89,90]. The faster migration of juvenile *O. keta* may be related to the extreme changes in water temperature along the East Sea. It is well known that increased water temperatures are associated with earlier downstream migration for juvenile salmonids [93]. Therefore, long-term surveys should be conducted to understand how the *O. keta* migration season is being impacted by climate change, which is affecting the coastal waters of the East/Japan Sea.

We were also able to detect the other three salmon species despite low eDNA concentrations. The population of other indigenous salmon, *O. masou*, was limited in the lower part of the Yangyangnamdae River [89], which supported our eDNA field test. Our study would be helpful to evaluate the natural population for this native species in the upper part of tributaries, since *O. masou* is more abundant in upstream of both tributaries and presented as a freshwater resident [90,94]. The detection of the two introduced species (*O. mykiss* and *O. kisutch*), which were imported from the United States [95,96], strongly supports their existence as established populations in the river system. Their continued monitoring will be important for the conservation of native salmon species. In particular, *O. mykiss* are among the farmed species near the river [97], and eDNA analysis of this species would be especially important to monitor any accidental introduction of farmed stock into the natural environment [98,99]. Long-term and standardized surveys for Pacific salmon species provide more sensitive and accurate information with respect to the spatiotemporal distribution, qualitative changes, or detection of introduced or alien species.

## 5. Conclusions

In summary, we successfully developed and optimized a new quantitative PCR assay to detect four *Oncorhynchus* species inhabiting the Korean rivers. This assay exhibited high sensitivity and specificity with both the low limit of detection (LOD) and the limit of quantification (LOQ) values. Field studies demonstrated that *O. keta* is most widely and abundantly distributed throughout the Yangyangnamdae River in both the spawning and non-spawning seasons, and is also the main migrating *Oncorhynchus* species in the river. Many small amounts of eDNA were identified for the other three species, which existed mainly in the mainstream during the non-spawning period. No significant quantitative difference between spawning and non-spawning seasons was identified for those three species suggesting that low numbers of these species are present in the river without migration. The identification of inhibition at varying rates from field samples suggests that eDNA copy numbers should be normalized in order to obtain more realistic and comparing values. Our study provides a standardized pipeline for the use of an eDNA-based quantitative PCR assay to monitor various aquatic organisms, not just salmonoid species. This will provide a more convenient and reliable method of monitoring populations than traditional programs currently in use in Korea.

## Figures and Tables

**Table 1:** Primers and probes used to detect four *Oncorhynchus* spp. in South Korea and the internal positive control (IPC) assay.

Species	Forward Primer (5′–3′) Reverse Primer (5′–3′) Probe (5′–3′)	Size (bp)
*Oncorhynchus keta*	CTACGGCTGACTAATTCGGAACATCCAC TCCTCACGGGAGGACGTAGCCC FAM-CGCCCGGGGACTTTATTACGGATCCTACCT-BHQ	187
*Oncorhynchus kisutch*	TTACACACCTCCAAACAACGAGGACTG TTGGCCGATAATGATGAATGGGTGTTCC FAM-CCCAATTCCTATTCTGGGCCTTGGTGGCG-BHQ	138
*Oncorhynchus masou*	GGGTTCTCTGTCGACAACGCCAC CTAAGGATGTTAGACAGAGAAGTATAGCTG FAM-CGTCATTACAGCTGCTGCAATCCTCCACCT-BHQ	229
*Oncorhynchus mykiss*	GAGGACTTTACTACGGCTCGTACCTC GTTAGAGTGGCGTTGTCAACGGAGAAG FAM-CTGCCTTTGTAGGCTACGTCCTCCCGTGAG-BHQ	229
*Zenarchopterus dispar* ^1^	CAGCAGCTATAAACGCATGAATTACAGG TTTTTGTCAGGTTGAGAGAATGAGTCCG	188

^1^ The IPC assay.

**Table 2:** The sensitivity and efficiency of four *Oncorhynchus* assays.

Species	LOD (Copy Numbers)	LOQ (Copy Numbers)	Efficiency (%)
*Oncorhynchus keta*	4.11	30	96.26
*Oncorhynchus kisutch*	8.44	45	95.15
*Oncorhynchus masou*	4.65	81	100.67
*Oncorhynchus mykiss*	10.38	34	106.33

## Data Availability

All the data is provided in the article and Appendix A.

## Appendix A

**Table A1:** Haplotype sequences retrieved from GenBank to construct a consensus sequence for designing primers and probes.

Species	GenBank Accession Number
*Oncorhynchus keta*	MN011557, MN011565, MN011555, MN011556, MN011562, MN011567, MK991795, MK991796, MK991797, MN011560, MN011558, JX960808, KU872716, MN011553, MN011561, MN011552, MN011564, KR778826, KR778827, KR778832, MN011563, MK991794, MN011554, MN011559, KR778834, NC_017838, KR778839, KR778838, KR778836, KR778845, KR778848, KR778849, KR778831, KR778833, KR778828, MN011566, KR778837, AF125212, FJ435616, FJ435617
*Oncorhynchus kisutch*	KU761856, JX185441, JX185442, KU761856, NC_009263, JX960809, MF621749, KP671851, JX960810
*Oncorhynchus masou*	JX960818, NC_008746, LC098718, FJ435612, NC_008747, FJ435611, NC_008745, JX960811, KY250421, LC098722, NC_009262, LC098720, LC098719
*Oncorhynchus mykiss*	AF125208, FJ435586, JX960814, KP013084, KU872710, AY032629, KU761858, AY032632, AY587174, AY587168, AY587172, AY587176, AF125209, MF621750, JX960815, DQ288271, AY032631, AY587169, FJ435599, NC_026537, FJ435595, AY587183, FJ435589, NC_001717, KP085590

**Table A2:** Quantitative PCR conditions for each salmon species.

		*O. keta*	*O. masou*	*O. mykiss*	*O. kisutch*
qPCR	Luna^®^ Universal Probe qPCR Master Mix (#M3004, New England Biolabs, Ipswich, MA, USA)	10 µL			
	Forward primer	0.4 µM	0.4 µM	0.4 µM	0.5 µM
	Reverse primer	0.4 µM	0.4 µM	0.4 µM	0.5 µM
	Probe	0.2 µM	0.2 µM	0.2 µM	0.25 µM
	eDNA	2 µL			
	ddH_2_O	Up to 20 µL			
qPCR cycles	Initial denaturation	95 °C, 5 min			
	Denaturation	95 °C, 20 s			
	Annealing	63.6 °C, 20 s	63.6 °C, 20 s	64.9 °C, 20 s	66.0 °C, 20 s
	Extension	72 °C, 20 s			
	Cycles	40			
